# Sources and Assembly of Microbial Communities in Vineyards as a Functional Component of Winegrowing

**DOI:** 10.3389/fmicb.2021.673810

**Published:** 2021-04-13

**Authors:** Reid G. Griggs, Kerri L. Steenwerth, David A. Mills, Dario Cantu, Nicholas A. Bokulich

**Affiliations:** ^1^Department of Viticulture and Enology, Robert Mondavi Institute for Wine and Food Science, University of California, Davis, Davis, CA, United States; ^2^USDA-ARS, Crops Pathology and Genetics Research Unit, Department of Land, Air and Water Resources, University of California, Davis, Davis, CA, United States; ^3^Department of Food Science and Technology, Robert Mondavi Institute for Wine and Food Science, University of California, Davis, Davis, CA, United States; ^4^Foods for Health Institute, University of California, Davis, Davis, CA, United States; ^5^Laboratory of Food Systems Biotechnology, Institute of Food, Nutrition and Health, ETH Zurich, Zurich, Switzerland

**Keywords:** viticulture, terroir, microbial ecology, microbiome, metagenomics, microbial dispersal, biogeography

## Abstract

Microbiomes are integral to viticulture and winemaking – collectively termed winegrowing – where diverse fungi and bacteria can exert positive and negative effects on grape health and wine quality. Wine is a fermented natural product, and the vineyard serves as a key point of entry for quality-modulating microbiota, particularly in wine fermentations that are conducted without the addition of exogenous yeasts. Thus, the sources and persistence of wine-relevant microbiota in vineyards critically impact its quality. Site-specific variations in microbiota within and between vineyards may contribute to regional wine characteristics. This includes distinctions in microbiomes and microbiota at the strain level, which can contribute to wine flavor and aroma, supporting the role of microbes in the accepted notion of terroir as a biological phenomenon. Little is known about the factors driving microbial biodiversity within and between vineyards, or those that influence annual assembly of the fruit microbiome. Fruit is a seasonally ephemeral, yet annually recurrent product of vineyards, and as such, understanding the sources of microbiota in vineyards is critical to the assessment of whether or not microbial terroir persists with inter-annual stability, and is a key factor in regional wine character, as stable as the geographic distances between vineyards. This review examines the potential sources and vectors of microbiota within vineyards, general rules governing plant microbiome assembly, and how these factors combine to influence plant-microbe interactions relevant to winemaking.

## Introduction

For thousands of years, wines have been made exclusively through autochthonous fermentations conducted by the microbiota (see Glossary) present in and on the fruit, or resident in the fermentation vessel (Chambers and Pretorious, 2010; Marsit and Dequin, 2015). *Saccharomyces cerevisiae* is the dominant species responsible for primary fermentation (alcohol production), but the transformation of grape must to wine is a multi-stage, multi-species process involving a diverse array of other microorganisms (Bokulich et al., 2016; Bisson et al., 2017; Hall et al., 2017). Enological starter cultures were first introduced into winemaking during the latter half of the 20th century (Chambers and Pretorious, 2010), yet uninoculated fermentations remain popular globally due to perceived benefits to wine quality, including regionality (Knight et al., 2015) and varietal character (Knight et al., 2018). Non-starter microbiota are involved in the fermentation of both inoculated and uninoculated wines, and must be introduced from one of two sources: either from the vineyard or from the winery (Bokulich et al., 2013).

**Figure 1:**
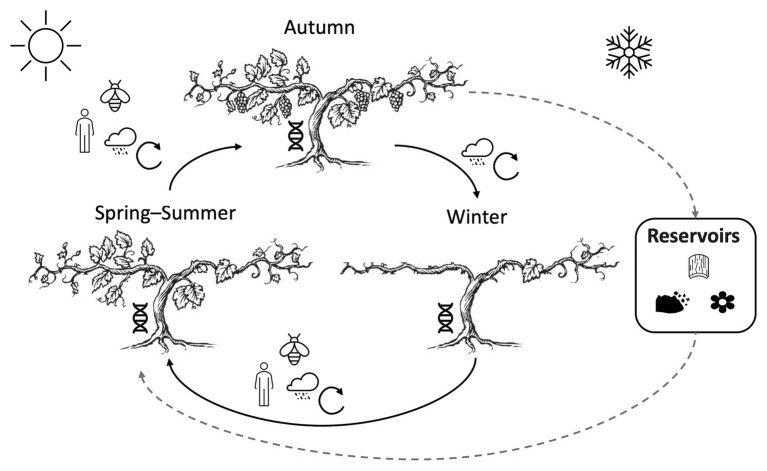
Spatial and temporal variation in vineyard microbiomes is shaped continuously by a mosaic of biotic and abiotic factors. Climate and weather patterns drive cyclical phenotypical stages of grapevines and their resident microbiota, spatial heterogeneity, and abiotic mixing/exchange of microbiota year-round (see also Figure 2). Several potential reservoirs (e.g., soil, grapevine bark, and other nearby plants) serve as overwintering sites for grapevine fungi and bacteria. Humans, insects, and weather events induce microbial transmission, particularly during the growing season (spring, summer, and autumn). Plant genotype continuously selects microbiota from this local pool.

The composition of grapevine-associated microbiomes – the fungi, bacteria, viruses, and other microorganisms inhabiting grapevines and their activities (see Glossary) – partly depends upon the vineyard location, cultivar, and farming method (Figure 1). These environmental factors all influence microbial effects on wine quality throughout the grape-to-glass continuum. For example, *Botrytis cinerea* and other grapevine pathogens exert pronounced, long-lasting effects on wine quality during grape development (Barata et al., 2012b; Blanco-Ulate et al., 2015, 2017; Martinez-Luscher et al., 2019). The potential impacts by specific microbiota derive from interactions among vineyard site, variety, and viticultural practices (Bokulich et al., 2014, 2016). Similarly, microorganisms exert both positive and negative effects on wine quality before, during, and following fermentation (Domizio et al., 2014, 2017; Blanco-Ulate et al., 2015; Belda et al., 2017; Hall et al., 2017; Reiter et al., 2021), spurring recent interest in the use of non-*Saccharomyces* yeasts in winemaking (Jolly et al., 2003, 2014).

Grapevine microbiomes exhibit spatial distribution between and within vineyards that correspond to environmental conditions, empirically defined viticultural zones, and regional wine properties (Setati et al., 2012; Burns et al., 2015; Bokulich et al., 2016; Knight et al., 2020). This connection between microbial biogeography (see Glossary) and regional wine characteristics has been termed “microbial terroir” (Bokulich et al., 2014; see also notes in Glossary), a term that hypothesizes a connection between grapevine microbiology and wine terroir (see Glossary). The evidence plays out in the empirical observations by many farmers, that grape and wine spoilage issues are often vineyard- and block-specific. Geographic distance is often a primary factor correlated with these differences, though it is unclear if it is merely a proxy for these other drivers (Burns et al., 2015; Miura et al., 2017). For example, microbiota in musts appear to correlate with vineyard and regional level climate and weather patterns (Bokulich et al., 2014; Reiter et al., 2021; Steenwerth et al., 2021), human activity, and human transport between vineyards in the case of *S. cerevisiae* (Goddard et al., 2010; Knight and Goddard, 2015; Gayevskiy et al., 2016). Site, encompassing the specific environmental constraints of a single place (e.g., vineyard or block), is commonly the most explanatory variable in studies of microbiome assembly in other plants, suggesting that vineyard-specific microbiomes should not come as a surprise (Knief et al., 2010; Rastogi et al., 2012; Coleman-Derr et al., 2016; Wagner et al., 2016). In this review, we characterize sources of microbiota in vineyards, specifically reservoirs, and the extent of transmission within and between vineyards. We assert that this serves as foundation for determining how suites of microbiota are related to wine characteristics, or even typicity, a stable regional or site specific wine signature.

Many questions remain unsettled regarding the microbiota within vineyards and their dispersal among vineyards and regions. Are soils or other plants the primary reservoir of microbiota that actually colonize (as opposed to temporarily inhabit) plant surfaces? Do yeasts present on the fruit surface in a previous vintage overwinter in fruit left behind, on the trunks of vines, in soils, or on neighboring vegetation? How is the fruit surface microbiome assembled annually, as fruit is a transient seasonal feature in vineyards?

To address these ideas, we examine microbial ecology, reservoirs, and transmission in vineyards from the ground up in an attempt to understand if and how environment, humans, and plant hosts together drive microbial assembly and interannual stability of grapevine microbiomes.

## Microbial Ecology of Vineyards: From the Ground Up

Before addressing the potential sources of microbiota in vineyards, we will establish baseline knowledge about the microbiomes found in the soil, on different plant organs, and the abiotic and biotic factors that shape microbiomes in these environments. Second, we will briefly summarize the importance of these microbial ecosystems on wine quality, to contextualize the importance of microbial source-sink relationships in vineyards.

Vineyard microbiomes (both in the soil and directly associated with grapevines) are shaped by multiple interacting factors that make up a single location, including climate (i.e., precipitation and temperature gradients), geolocation, elevation, topography and slope, edaphic factors, and management practices for the soil and the grapevine (Barata et al., 2012b; Bokulich et al., 2014; Burns et al., 2015, 2016; Jara et al., 2016; Portillo et al., 2016; Vitulo et al., 2019; Liu et al., 2020; Steenwerth et al., 2021). Microbial biodiversity in the soil, grapevine, and surrounding environments thus reflect effects of both environmental filtering and dispersal limitation (see Glossary), as detailed below.

### Vineyard Bulk Soils

The functional activities of soil microbiota are integral to biogeochemical cycles, and directly impact soil fertility and chemistry (Kallenbach et al., 2016; Fierer, 2017). Soil microbiota interact with plants in the rhizosphere, the soil zone directly surrounding plant roots, and influence plant health, physiology, and phenotype (Wagner et al., 2014; Huberty et al., 2020). Distinct from work on plant-microbe interactions (Berg et al., 2014; Trivedi et al., 2020) and examinations of bulk soil and rhizosphere microbiota (Fierer, 2017; Fitzpatrick et al., 2018), we focus on relationships between grapevines and soil microbiota, their potential impacts on vine physiology and grape chemistry, and potential transmission of fungi and bacteria from soil to the grapevine.

Biogeographic patterns in soil microbiomes from vineyards and other land use types have been revealed at multiple spatial scales, including continental, region, site, and even within sites (Fierer and Jackson, 2006; Schreiner and Mihara, 2009; Drenovsky et al., 2010; Martiny et al., 2011; Fierer, 2014, 2017; Holland et al., 2014; Talbot et al., 2014; Wagner et al., 2014; Burns et al., 2015; Zarraonaindia et al., 2015; Liang et al., 2019). Both dispersal limitation and environmental filtering (see Glossary) explain patterns of microbial biogeography. In soils, drivers include static properties, such as mineralogy, morphology, texture, and pH, whereas more dynamic soil properties include fluctuations in water content and temperature, and resource availability derived from quantity and quality of carbon and nitrogen (N) pools (Burns et al., 2015). Like other land use types, bulk soil bacterial community composition in vineyards corresponds to shifts in soil pH and C:N ratio in the fine fraction across soil types (Burns et al., 2015; Zarraonaindia et al., 2015). Together, these factors affect the environmental conditions acting on soil microbiota, and in turn, microbiota act as architects of their own environment through nutrient transformations, exudation of mucilages, and formation of soil aggregates. These activities by microbiota then influence the diffusion and movement of resources through the soil. Soil management practices also modulate bulk soil microbiomes, as practices like cover crops and cultivation alter resource availability and edaphic factors like soil pH (Burns et al., 2016; Chou et al., 2018; Pingel et al., 2019). Thus, local soil characteristics are the primary driver shaping the extant pool of microorganisms that interact with grapevines, but geographic distance can also be related to differences in soil microbiomes.

The distance-decay relationship describes the similarity in species composition between two or more communities with respect to the distance between them, with increasing dissimilarity across increasing distance serving as a reflection of dispersal limitations in microbial communities across various spatial scales. For example, fungal soil communities in vineyards show site specificity at the local scale (within 2 km), and increasing distances between sites are correlated with increasing soil microbiome diversity at the large scale (>100 km; Morrison-Whittle and Goddard, 2015; Knight et al., 2020). Dispersal limitations also drive soil microbiome composition and structure, as historical contingencies (or previous dispersal events and geographic isolation) facilitate speciation of individual microbiota and further structuring of microbiome membership (Talbot et al., 2014; Peay et al., 2016).

Although membership of a microbial community can be driven by dispersal limitation, leading to a high degree of spatial heterogeneity of soil microbiomes, functional redundancy can lead to partial decoupling of taxonomy and function. Functional redundancy describes the shared metabolic functions among taxonomically distinct, coexisting community members (Louca et al., 2018), and can be extended to describe microbiomes occupying similar niches that are taxonomically distinct but perform similar metabolic functions, such as nutrient cycling. Thus, spatial variation in species compositions (e.g., in vineyard soils) does not necessarily amount to spatial variation in core functions, such as nutrient cycling. Nevertheless, evolutionary relatedness and function are partially linked (Zhu et al., 2015), and hence the importance of microbial biodiversity (in terms of species and functions) cannot be understated.

### The Rhizosphere: A Primary Plant-Microbial Interface

The rhizosphere describes the zone of soils that contact plant roots and are influenced by root exudates, wherein plants and soil microbes most closely interact (Mendes et al., 2013; Lazcano et al., 2021). The rhizosphere microbiome of grapevines (as with other plants) is impacted by hierarchically structured relationships between geographic location, plant genotype, and edaphic factors including land use history (Berg and Smalla, 2009; Wagner et al., 2014, 2016; Kaplan et al., 2020). As potentially long-lived woody perennials, grapevines have an extended opportunity to form lasting relationships with microbial communities. Variations in root morphology and exudates allow plants to actively recruit rhizosphere microbiota from among the general population of microbiota in the surrounding soil (Berg and Smalla, 2009; Tkacz et al., 2015), leading to an orders-of-magnitude reduction in microbial diversity from bulk soils to the rhizosphere to the root surface. This process is influenced by vine age as hosts continue to exert selective pressure over long time periods (Holland et al., 2014), and by the scion and rootstock genotypes as different cultivars recruit different microbiota from similar pools (D’Amico et al., 2018; Marasco et al., 2018; Berlanas et al., 2019). Plants exude 10–44% of their photosynthetically derived carbon to the rhizosphere and communicate with microbiota through hormonal signaling and production of volatile organic compounds (VOCs) (Berg and Smalla, 2009; Mendes et al., 2011). As sessile and even long-lived organisms, plants partly engineer their local soil environment through active rhizosphere selections (Canarini et al., 2019).

The rhizosphere serves as a microbial extension of the hosts’ metabolic and genomic repertoire, analogous to the role of gut microbiomes to, e.g., human health (Bulgarelli et al., 2012; Hacquard et al., 2015), and hence plants actively modulate their rhizosphere microbiota to cultivate beneficial symbionts. Rhizosphere microbiota play pivotal roles in nutrient acquisition, growth, and development of plants (Mendes et al., 2013; Tkacz et al., 2015), alteration of root architecture (Vacheron et al., 2013; Rolli et al., 2016), timing of phenological stages (Wagner et al., 2014), acquisition of trace metals and mineral nutrients (Baldan et al., 2015; Lazcano et al., 2021), drought tolerance (Coleman-Derr et al., 2016; Vurukonda et al., 2016), and defense against biotic stressors such as pathogens (Mendes et al., 2011; Lazcano et al., 2021). Rhizosphere microbiota can induce systemic resistance to plant pathogens, and be recruited in response to foliar pathogens, producing persistent effects in soil through production of root exudates and affecting successive generations of plants grown in the same soil (Berendsen et al., 2018; Yuan et al., 2018). These findings reveal that biotic and abiotic stressors, land use history, and agricultural practices create a biotic legacy in shaping the functional structure of the rhizosphere microbiome.

In grapevines, rhizosphere microbiota can enhance drought resistance in multiple rootstock varieties (Salomon et al., 2014; Rolli et al., 2015). Alleviation of water stress in grapevines can occur through promotion of plant growth by *Bacillus* and *Pseudomonas* species, as they induce production of abscisic acid (ABA) by the grapevine (Salomon et al., 2014). Importantly, carefully timed water stress is a commonly used farming technique to induce production of desirable secondary metabolites, specifically phenolics in red wine grapes (Roby et al., 2004), and water stress is related to bud fertility (Guilpart et al., 2014). Could there be a potential interaction between anthropogenic activity (induced water stress), rhizosphere microbiota recruitment, and grapevine phenotype (secondary metabolite production), as has been shown in other plants (Baslam and Goicoechea, 2012)?

Microbial interactions with grapevines at the root-soil interface also have the potential to shape wine qualities through changes in fruit chemistry. Functional changes in drought tolerance (Salomon et al., 2014; Rolli et al., 2015; Coleman-Derr et al., 2016; Vurukonda et al., 2016), the uptake of nitrogen and other trace minerals such as phosphorus (Mendes et al., 2013; Baldan et al., 2015; Trouvelot, 2015), and timing of phenological stages (Wagner et al., 2014) are all ways that soil microbiota might affect the production of wine-relevant metabolites by grapevines as demonstrated in other plants. These effects of microbial interaction with rhizosphere microbiota could underpin some of the phenotypic plasticity seen by single grapevines cultivars at different sites (Wagner et al., 2014). Arbuscular mycorrhizal fungi have been shown to alter the uptake of N in grapevines, spurring biomass accumulation (Cheng and Baumgartner, 2004; Trouvelot, 2015). Nitrogen uptake alters the partitioning of resources between plant biomass and fruit development, as well as secondary metabolite production in grapevines (Habran et al., 2016). Furthermore, N content in grapes provides nutrients to microbiota during fermentation, and hence is linked to yeast viability and metabolite production during fermentation (Bell and Henschke, 2005). Acquisition of mineral nutrients by the grapevine also has the potential to alter the fermentation kinetics of musts through changes in redox potential (Killeen et al., 2018).

### Aboveground Plant Compartments

Grapevines exhibit complex physiology and phenology, with multiple aboveground compartments (the “phyllosphere,” see Glossary) with distinct selective conditions for microbial growth, principally the leaves, bark, and fruit (the “carposphere,” see Glossary). These are spatially related but functionally distinct plant compartments, and are colonized by distinct microbial communities (Morrison-Whittle and Goddard, 2018).

Among all of these compartments, fruit is the only compartment in which the microbiota present can be directly implicated in wine outcomes. Microbial activities on fruit can be strongly influential to wine quality long before harvest, as most clearly exemplified by fungal growth that can cause undesirable (Barata et al., 2012b) or desirable wine characteristics (Bokulich et al., 2012b; Blanco-Ulate et al., 2015). Selection of microbes on the grape surface might likewise influence susceptibility to grapevine pathogens through microbe-microbe interactions between primary colonists and future pathogens (Agler et al., 2016; Berg and Koskella, 2018), and theoretically could influence colonization patterns more generally, e.g., of fermentative yeasts. The microbiota present on the grape surface at harvest also serve as the initial fermentation consortium present in early wine fermentation. Other plant compartments are unlikely to select for strongly fermentative organisms, as they lack the selective conditions of fruit (namely, low pH and a concentrated source of sugars), but a fair amount of bark and leaves can become intermixed with grapes during harvest and should be thoroughly considered in this discussion as potential reservoirs and vectors for fermentative microorganisms, as recent reports suggest that they could be a reservoir for such microbiota (Morrison-Whittle and Goddard, 2018; Nadai et al., 2019).

#### Plant Compartment Drives Microenvironment Colonization

Plant compartments harbor distinct microbial communities, as niche effects (see Glossary) exert selective pressure within sites (Martins et al., 2013; Zarraonaindia et al., 2015; Coleman-Derr et al., 2016). In grapevines, plant compartments exert stronger influence on community structure than geographic distance (Morrison-Whittle and Goddard, 2015) or site (Zarraonaindia et al., 2015). However, vineyard/site is highly explanatory of microbiome composition when constraining the analysis by plant compartment in grapevines (Morrison-Whittle and Goddard, 2015; Zarraonaindia et al., 2015) as well as in other plants (Coleman-Derr et al., 2016; Wagner et al., 2016).

Niche effects structure highly distinct microbiomes between plant compartments, while there is often overlap in membership within a site or individual vine (Morrison-Whittle and Goddard, 2015, 2018; Coleman-Derr et al., 2016; Cregger et al., 2018). This does not discount the effect of biogeography on structuring microbiomes in vineyards, but highlights the importance of niche and selective forces in defining microbiome structure in individual plant compartments. Plant compartments select for microbial species that are evolved to inhabit that niche, but environmental filtering and dispersal effects act as a primary filter, shaping the local pool of microbiota that can colonize plant compartments.

#### Plant Genotype: The Third Wheel Driving Microbiome Selection

Emerging evidence suggests that the relative importance of drivers may be hierarchically structured: in the case of grapevines, host genotype and niche or plant compartment appear to be secondary to site-driven effects on grape microbiome composition, and host differences appear stronger within regions or sites (Bokulich et al., 2014; Portillo et al., 2016; Wagner et al., 2016).

Host genotype exerts an effect on the microbiota present in the phyllosphere of various plants. This occurs through production of antimicrobial compounds and selection of hub taxa (see Glossary) that affect downstream community development through microbe-microbe interactions, and through the morphological characteristics of their vegetative and then sexual structures (Bodenhausen et al., 2014; Agler et al., 2016; Wagner et al., 2016). Microorganisms in the phyllosphere are found near structural features of leaves like veins and stomata, embedded in multi-species biofilms (Vorholt, 2012). These physical niches represent microsites that participate differentially in the release of nutrients (Vorholt, 2012), as well as in modulating stressors encountered at the plant surface microenvironment (e.g., UV and water availability). Grapevine cultivars are differentiated by morphological characteristics that alter the leaf microenvironment, including cuticle profiles, cluster compactness, and cluster and leaf morphologies (Gabler et al., 2003; Chitwood et al., 2014; Tello and Ibáñez, 2018). The combination of these characteristics impact cultivar susceptibilities to fungal pathogens like *B. cinerea* (Gabler et al., 2003; Herzog et al., 2015; Tello and Ibáñez, 2018). Similar impacts can be expected for phyllosphere microbiomes at large.

Differences in microbiota with respect to grapevine cultivar are likely driven by the aforementioned factors, specifically plant morphology and physiology. The extent of these effects is unclear, due to covariation between cultivar selection, climate, and management practices. Planting grapes in single cultivar blocks (as is standard practice) limits parsing of the relative effect of site and cultivar on associated microbiota (Bokulich et al., 2014). Furthermore, cultivars are typically managed according to their growth patterns, including canopy training system, which influences canopy microclimate. However, microbiome variation with respect to cultivar is consistent across geographic regions in grape musts, suggesting a host-genotype mediated selection of specific taxa in the carposphere (Bokulich et al., 2014). Determining if microbiome assembly corresponds to genotype, as seen in other plants, will require comparison between cultivar phylogeny and community assembly with sufficient experimental replicates and careful design. Such efforts may be critical to future precision management strategies, and farming for varietal and regional wine typicity.

#### Site Effects: A Combination of Environment and Microbial Sources

The phyllosphere microbiome results primarily from site effects, representing a combination of environmental filtering by abiotic factors, dispersal limitation, and subsequent species filtering by host plants. As in soils, there may be larger differences within vineyards than between, and accordingly, scale and proper number of samples are critical to drawing ecological conclusions (Burns et al., 2015; Zarraonaindia et al., 2015). Microbes are differentially dispersed due to their life-strategies and morphological features, and environmental factors shape the regional pool of microorganisms as reservoirs or through altering dispersion. These include regional weather patterns that drive dispersion and deposition like wind and rain (Madden, 1997; Bokulich et al., 2014), the interaction of these weather patterns with landscape scale features (Mahaffee and Stoll, 2016), proximity to microbial point sources like other farms or roads (Bowers et al., 2011), landscape connectivity between vineyards (Meentemeyer et al., 2012), vectoring by insect hosts (Stefanini et al., 2012; Madden et al., 2017; Quan and Eisen, 2018), and the local environment, including surrounding soil, permanent compartments of perennial plants, neighboring plants, and surrounding forests (Fort et al., 2016). These reservoirs and vectors are considered in more detail later in this review.

Microbial communities colonizing grapevine compartments change in composition throughout the growing season in response to phenological and environmental changes (Martins et al., 2012). Grapevine microbiomes also exhibit interannual variation (probably in response to weather), but remain semi-stable between regions and across vintages (Bokulich et al., 2014; Cheng et al., 2020; Reiter et al., 2021). How do we account for these findings in understanding the microbial source-sink relationships between vines and the site-defined regional pool? Current analyses point to soil, air, insects, and plant compartments as potential reservoirs for microbial colonists inter-annually (Knief et al., 2010).

## Mechanisms of Microbial Dispersal and Potential Sources

**Figure 2:**
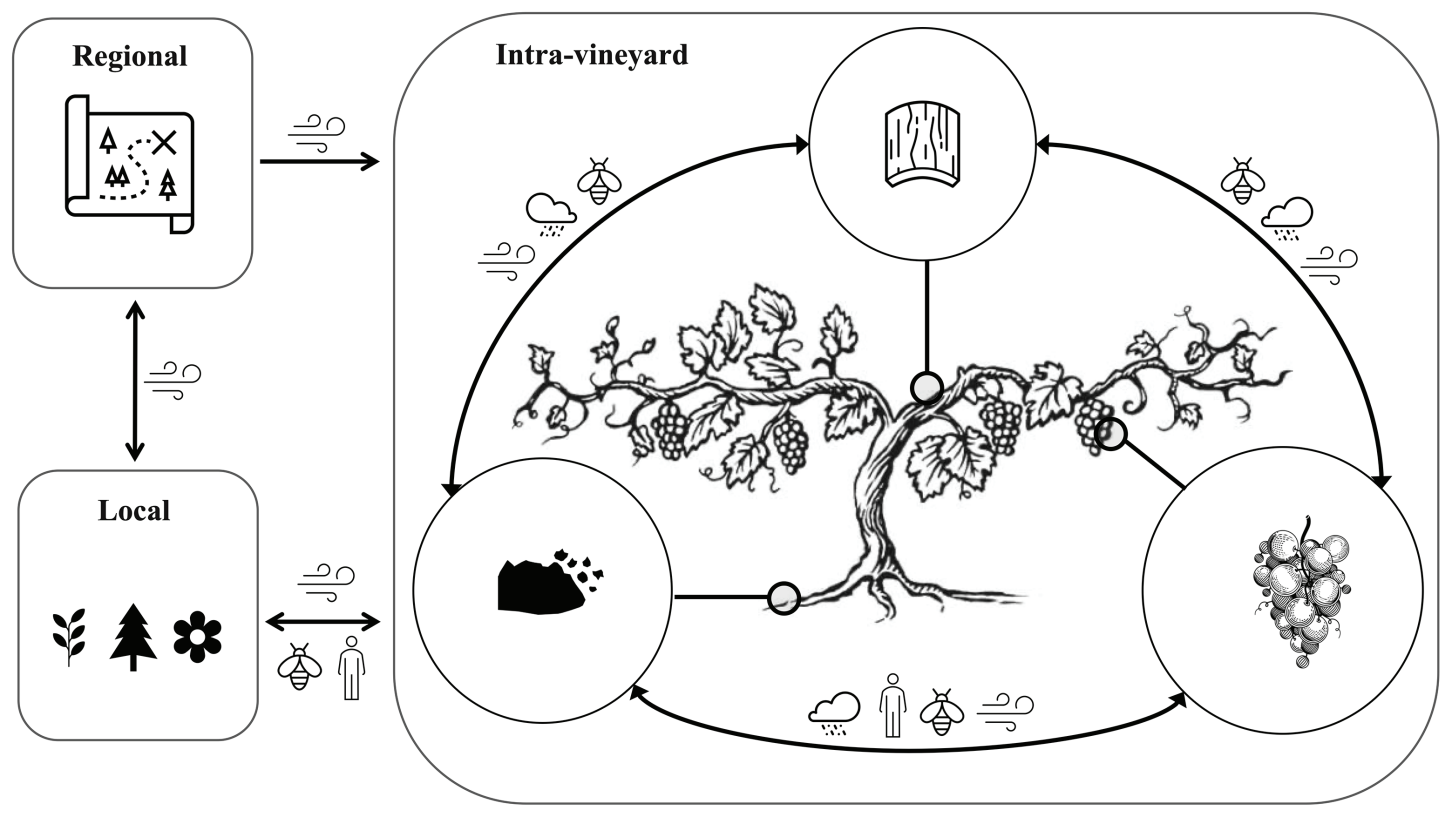
The vineyard is an interconnected and open ecosystem that exchanges microbiota at intra-vine, intra-vineyard, local, and regional scales. Microbiota are naturally exchanged between the regional, local, and intra-vineyard scale by wind/weather factors, and locally by human activity, insects, and other factors. At the intra-vineyard and intra-vine scales, microbes are exchanged between vines and plant compartments (grapes, phyllosphere, and rhizosphere/soil) by various vectors, including wind, rain, insects, and human activity. These transmission pathways are subject to dispersal limitation, shaping the local pool of available microbiota. Environmental factors and plant genotype exert further selective pressures to shape the microbiota of different plant compartments.

Phyllosphere microbiomes are assembled annually in temperate climates, and exhibit seasonal cyclicity in microbial colonization (Figure 2). This cyclicity is particularly pronounced in fruit, as seasonally ephemeral organs that select a compositionally and functionally distinct microbiome, including osmophilic (and fermentative) yeasts. It is unclear whether these microbial colonists overwinter in soils, plants, or other protective compartments (e.g., neighboring wineries; Viel et al., 2017; Chalvantzi et al., 2020), and how they are vectored to the surface of the nascent vegetative or reproductive structures of the plant. The potential for soils, neighboring plants, wind, and permanent grapevine structures like the trunk for harboring organisms of clear importance to wine quality are examined in this section. Vineyards are open, interconnected ecosystems, highlighting the importance of system-wide stewardship for plant health and wine quality outcomes.

### Soils as a Reservoir for Phyllosphere Microbiota?

Soils have repeatedly been argued to be the primary reservoir for microbiota on plant surfaces aboveground, including in grapevines (Martins et al., 2013; Zarraonaindia et al., 2015; Wagner et al., 2016; Figure 2). Conceptually, the idea of phyllosphere community members originating atleast in part from soils is not challenging to imagine, but is difficult to systematically investigate (Fort et al., 2016; Chou et al., 2018). Microorganisms that are putatively derived from the soil are distributed in both the endosphere and the phyllosphere of grapevines and other plants (Compant et al., 2008; Zarraonaindia et al., 2015; Coleman-Derr et al., 2016; Wagner et al., 2016; Morrison-Whittle and Goddard, 2018), though the direction of transmission is difficult to establish. Alternative explanations for the co-occurrence of microbiota in the soil and phyllosphere include that microbes present on fruit or leaves are shed onto the soil surface and detected at the time of sampling, or that the microbes in both the phyllosphere and soil are deposited from some third source.

The idea of shared taxa between soils and vines in winegrowing was established by Martins et al. (2013) and Zarraonaindia et al. (2015), with regard to bacterial communities. Both authors found a reduction in bacterial diversity from soils, to bark, to fruit and leaves, and proposed tillage as a mechanism by which soil microbiota could be unintentionally vectored onto grapes (Martins et al., 2013; Zarraonaindia et al., 2015). The same relationship has also been shown for fungi (Morrison-Whittle and Goddard, 2015, 2018). Needless to say, the soil is a persistent reservoir of many of the microorganisms that are also found on nearby plants, but the more important question is whether the microorganisms with important roles for plant health (and in the context of this review, wine quality) can survive for substantial periods of time in soils. In some cases, soil is clearly a reservoir for plant-associated microbiota: *Burkholderia* species actively gain entry into grapevines through roots and make their way sequentially through the plant organs (Compant et al., 2008). However, actual visualization of vectoring and determination of sources and sinks for other microorganisms have yet to be demonstrated (Fort et al., 2016; Morrison-Whittle and Goddard, 2018). More recent work has shown that soil management impacts soil microbiota but not grape microbiota, contradicting the idea that grape microbiota are responsive to soil microbial sources directly under the vine (Chou et al., 2018).

Whether soil acts as a reservoir for the primary fermentation-relevant microbiota (e.g., fermentative yeasts) is even more unlikely and difficult to prove. Soil and fruit surface environments pose vastly different selective conditions for microbiota. If fruit and soil microbiomes are similar at the point of sampling, this may just represent the fact that vineyards are dusty, tractors move dust, and dust is moved by the wind. To date, no study has tracked specific microbial strains from vineyard soils to grape musts or wines. The occurrence of fungal microbiota in vineyard soils, surrounding forests, and grape musts has been demonstrated, though the direction of transmission has not been proved (Morrison-Whittle and Goddard, 2018). Late in the growing season, fruit is often thinned and dropped to the ground, presenting a potential source for wine-relevant yeasts on the fruit and in soils. The question is whether these species can survive long-term (e.g., overwinter) in the soil itself. Fruit and plant detritus appear to be important reservoirs for fermentative yeasts (Sipiczki, 2016) and a more likely reservoir than soil itself. On the other hand, soil has recently been shown to induce sporulation in *S. cerevisiae* (Knight and Goddard, 2016), one means by which long-term survival of this yeast could be accomplished during periods of nutrient limitation (e.g., when fruit is not present).

Similarities between phyllosphere and soil microbiota could be driven by frequent mixing of vineyard microbiota by wind and rain, rather than transfer of soil-derived microbial colonists that are well-adapted for life in the phyllosphere. These natural forces vector soil-borne microbiota at different scales (Madden, 1997; Bowers et al., 2011; Bock et al., 2012; Albright and Martiny, 2018), and facilitate bidirectional exchange of microbiota between soils and plant surfaces (Madden, 1997). The spatial distance between soil and the fruiting zone determined by grapevine training style likely alters the deposition of soil microbiota on fruit. However, whereas local soil microbiomes are structured in response to edaphic factors (Burns et al., 2016), and undervine management alters fungal microbiomes (Chou et al., 2018), these effects are not reflected on the adjacent fruit surface. Thus, wind and rain are likely the prevalent abiotic mechanisms of microbial exchange in vineyards, but the questions of source, sink, and long-term survival remain unresolved.

### Grapevine Endophytes: Hitchhikers From Soil to Grapes?

The endosphere refers to all internal tissues of the plant. While internal plant tissues are generally not as microbially complex as external tissues, they are not sterile (Coleman-Derr et al., 2016). Endophytes – microbes living within plant tissue – exist naturally in many grapevine organs, primarily in the roots and vasculature in healthy plants. Endophytes and epiphytes may be part of a continuum, as microbiota existing in the rhizosphere or phyllosphere can gain entry to the vasculature and become endophytes (Bulgarelli et al., 2012; Coleman-Derr et al., 2016).

Migration from the rhizosphere is a prominent access point for microbiota found in the grapevine endosphere. Compant et al. (2005) demonstrated that *Burkholderia* sequentially colonized roots before moving through vasculature, where it was found in distal substomatal chambers, and not the outer surfaces of the leaves (Compant et al., 2005). This was first shown in a gnotobiotic grapevine model (Compant et al., 2005), then in non-sterile soils (Compant et al., 2008). Further investigation revealed that viable *Pseudomonas* spp. and *Bacillus* spp. in the xylem of flower ovules and the internal structures of pulp cells in berries, suggesting that these represented phyllosphere colonists based on their localization in the endosphere (Compant et al., 2011). Thus, the grapevine endosphere is naturally colonized through both below- and above-ground routes, and there could be distinct root and leaf endospheres within the same plant, as shown in other plants (Coleman-Derr et al., 2016).

Microorganisms can also access the grapevine endosphere through above-ground organs, leading to pathogenesis. For instance, *B. cinerea* commonly exists as a fungal wind-borne pathogen, and upon transmission to flowers, exists as a latent endophytic infection in developing grapevines (Haile et al., 2017). Fungal pathogens can gain entry through pruning wounds, and exist as multi-species disease complexes such as in the case of *Esca* (Morales-Cruz et al., 2018). Insect pests can also transmit microbiota into the endosphere, including *Xylella fastidiosa*, the causative agent of Pierce’s disease (Lopez-Fernandez et al., 2017).

More recently, it has been hypothesized (but unproven) that fermentative organisms from the soil may end up as endophytes in grape berries, transmitted through the xylem (Liu et al., 2020). Diverse microorganisms have been detected in surface-sterilized berries, including non-Saccharomyces yeasts and *S. cerevisiae* in at least one instance, though these findings should be replicated, and the point of entry is unclear (Hall and Wilcox, 2018). Taken together, these findings represent a potentially paradigm shifting possibility for microbial seeding of musts, and should be further explored as a component of the system-level impact of microbial diversity among sites on wine outcomes.

### Dispersion and Deposition by Wind

Air is replete with microbiota, transported in aerosols (Womack et al., 2010) and dispersed via air currents on regional and continental scales (Kellogg and Griffin, 2006; Schmale and Ross, 2015). Movements on this scale have relevant consequences for plant health: for example, *Plasmopara viticola* (the causative agent of grape downy mildew) can traverse the eastern seaboard of the United States in a single growing season (Schmale and Ross, 2015).

On the regional scale, microbial communities in the near-surface atmosphere are structured with regard to land use (e.g., urban vs. agricultural) and season (e.g., responsive to plant growth; Bowers et al., 2013). Changing meteorological conditions do not appear to explain this seasonal variation, as bacterial community structure in near-surface atmosphere is related to land use type, not local weather (Bowers et al., 2011). Moreover, wind is implicated in the seeding of phylosphere microbiomes, as they mirror local airborne microbiomes initially (Maignien et al., 2014).

On the local scale, dispersal limitation effects are evident, but landscape features play a more prominent role in shaping microbial dispersion patterns by interacting with air currents. These patterns occur though both habitat connectivity at the inter-vineyard or landscape scale (Meentemeyer et al., 2012), and are apparent at the farm-scale as gradients across orchards (Theofel et al., 2020) and variable intra-vineyard distribution of organisms (Madden, 1997; Bock et al., 2012; Mahaffee and Stoll, 2016). As an example, local dispersion of *Erysiphe necator* (the causative agent of grape powdery mildew) by wind is influenced by landscape connectivity and heterogeneity (Meentemeyer et al., 2012). Wind dispersion may connect microbial ecosystems (multiple microbiomes within a larger geographic land-use system) at the local scale, and hence microbial dispersion may be influenced by local landscape features and land use.

At the scale of vineyard (site), wind and rain can disperse fungal pathogens (and other microbes) on the order of meters between individual plants and soils (Madden, 1997; Bock et al., 2012; Mahaffee and Stoll, 2016; Haile et al., 2017). Row orientation, spacing, and canopy height affect the dispersion of the fungal pathogen *E. necator* (Bailey and Stoll, 2013), by altering air circulation patterns among vines. Heterogeneity in trellised canopies decreases the ability of particles to escape, and particulates move down rows biased by wind direction resulting in instances of powdery mildew infections extending down vineyard rows (Bailey and Stoll, 2013; Bailey et al., 2014; Mahaffee and Stoll, 2016). Training style of grapevines and row spacing thus likely alter the microbiota present due to their effects on airflow and proximity to the ground.

Taken together, these findings suggest wind is a relevant vector for seeding and distribution of vineyard phyllosphere communities. The specific location, layout, and landscape-scale features of a vineyard may result in seasonally stable microbiomes, and interannually stable dispersion patterns both within and between vineyards by subtly influencing the exchange rate of microbiota between soils, plants, and the surrounding airspace.

### Land Use and Vegetation as Reservoirs for Vineyard Microbiota

Surrounding land can be another reservoir for microbial colonists of vineyards, vectored either by wind or insects. Fungal species (Hyma and Fay, 2013; Dashko et al., 2016; Fort et al., 2016; Castaneda and Barbosa, 2017) and strains (Knight and Goddard, 2015) are shared among surrounding forests, vineyards, and grape musts (Morrison-Whittle and Goddard, 2018). Similarly, wild *Vitis* species in unmanaged habitats surrounding vineyards could serve as reservoirs for beneficial and pathogenic microbiota that might be vectored across habitats like vineyards and the surrounding environment by insects (Baumgartner and Warren, 2005; Kernaghan et al., 2017).

Plant genotype-mediated recruitment of microbial symbionts may influence the resultant local pool of microorganisms, reflecting the genetic mixture of grapevines, cover crops, weeds, and other local vegetation. Neighboring plants serve as potential reservoirs for microbiota that are already adapted to the pressures of life in the phyllosphere, including UV radiation, while vineyard weeds harbor endophytic microbiota with distinctions by plant genotype (Bulgarelli et al., 2013; Samad et al., 2017). Wind and other site-dependent vectors may regulate the degree of mixing of phyllosphere microbiota within the “neighborhood” in a site-specific fashion. Anecdotally, some winegrowers speak of local vegetation being a feature of their terroir, and molecules from neighboring plants are known to be detectable in wines (Poitou et al., 2017). If neighboring flora donate their flavors to the grapes, could they also donate their microbiota to the vineyard community, altering grape and wine qualities by contributing to the local mosaic of microbiota?

### Insects as Vectors

Evidence from studies of insect-driven yeast dispersal are helping shape new frameworks to explain microbial community ecology, and suggest that their activity is integral to shaping seasonal microbiome assembly in plants (Chappell and Fukami, 2018; Madden et al., 2018; Toju et al., 2018). Two recent hypotheses describe the relationship between seasonally ephemeral plant sugar sources (e.g., fruit and flowers) and the vectoring of yeasts by insects. Both could support interannual dynamics of yeast populations in vineyards and microbial terroir, and influence on the annual assembly of grapevine phyllosphere and carposphere microbiomes.

The “fruit forest-reservoir” hypothesis attempts to explain the interannual presence of *S. cerevisiae* on the ephemeral fruit in vineyards via vectoring by insects between soils and fruit (Knight and Goddard, 2016). In this proposed model, *S. cerevisiae* (and by extension many other yeasts) is posited to exist in substantial numbers on damaged fruit deposited in the vineyard (Knight and Goddard, 2016). Soil contact induces sporulation in *S. cerevisiae*, facilitating overwinter survival as spores prior to insect vectoring to fruit in the Spring (Knight and Goddard, 2016). The key to this hypothesis is the overwintering of yeasts in soils, but insects are an important component of the annual transmission cycle.

The “dispersal-encounter hypothesis” broadly describes the ecological relationship between yeasts and insects, and the yeast communities in flower nectar (Madden et al., 2018). This hypothesis posits that both parties (microbial and insect) gain fitness from this relationship: insects disperse yeasts to ephemeral, seasonal, and spatially separate sugar sources, and insects – such as wasps, bees, and flies – use specific volatile metabolites produced by fermentative yeasts as signals to find sugar sources (Buser et al., 2014). Both wind and insects (interacting with visual cues from flowers) initially transport yeasts to the fruit surface, and a variety of fermentative yeasts produce volatile metabolites that attract insects (Quan and Eisen, 2018; Jones et al., 2021). Yeasts benefit from this relationship with insect vectors by arriving to new seasonal sugar sources before competitors, and once there can engineer the environment through the production of ethanol (Goddard, 2008; Madden et al., 2018). This is relevant to winegrowing: drosophilids and wasps in the vineyard during ripening are known to harbor yeasts that differ by vineyard location, reflecting their interactions with the local environment (Lam and Howell, 2015; Sipiczki, 2016).

Aside from aiding in their distribution in space, insects also disperse yeasts through time (Madden et al., 2018) and support the interannual persistence of yeast strains within a vineyard. Queens of the social wasps *Vespa crabro* and *Polistes* spp. can harbor *S. cerevisiae* cells while overwintering and transmit cells vertically to their offspring (Stefanini et al., 2012). These wasps both consume and propagate *S. cerevisiae* in the vineyard, actively feeding on grapes and break the skin to access sugars.

Morphological and molecular features in both insects and yeasts reflect their evolutionary mutualism and support their continued ecological connections. For example, extra layers of chitosan on *Saccharomycetales* yeasts and clumped spores from *Metschnikowia gruessi* allow them to stick more easily to fine hairs of insects (Madden et al., 2018). Transit through the guts of insects also facilitates hybridization of yeast spores, leading to outbreeding of yeasts (Madden et al., 2018). Thus, the mutualism between wasps and yeasts may play a role in development of new genotypes (Knight and Goddard, 2016).

These findings reveal that insects serve as vectors for wine-relevant fungal taxa in the vineyard and winery environment. Further, microbial transmission by insects is not restricted to *S. cerevisiae*, and the contingent microbiome of insects reflects their source environment. In principle, any insect feeding on a grapevine’s vegetative or fruit tissues could shape the associated microbial communities. Insects also visit *Vitis vinifera* flowers (Hogendoorn et al., 2016), thus serving as a potential source of seed microbiota that could inoculate plant organs emerging at different phenological stages of plant development. Together, the evidence supports a complex interconnection between fructivorous insects, yeast, grapevine health, and wine quality.

### Microbial Exchange Between Plant Compartments

Exchange of microorganisms between plant compartments offers another hypothetical reservoir for interannual assembly of grapevine microbiomes. Grapevine bark could be a reservoir for microbiota that re-colonize other grapevine compartments during the growing season. Viable *Uncinula necator* cleistothecia over-winter in grapevine bark fissures and on senesced leaves (Grove, 2004). Initial powdery mildew infections in the following season were found on the abaxial sides of leaves, nearest to exfoliating bark of heads and cordons of vines, suggesting that these were the sources of primary inoculum for the vineyards (Grove, 2004). Similar findings have been made in the model woody perennial *Populus populus*, suggesting bark as a site for overwintering of the resident microbial reservoir that might migrate to the vegetative tissues by wind, rain, or insect vectoring (Cregger et al., 2018). Substantial taxonomic overlap has been documented between vineyard soil, bark, fruit, native forests, grape juice, and wine fermentations, further highlighting the potential role of bark as a microbial reservoir in vineyards (Morrison-Whittle and Goddard, 2018).

Leaves can be another reservoir for microbiota during the growing season. Leaves emerge first in the seasonal grapevine cycle, and at the time of veraison, the intact berry surface microbiome resembles the grape leaf microbiome, dominated by Basidiomycetous yeasts and *Aureobasidium pullans* (Barata et al., 2012b). This could suggest that leaves are a reservoir for microbiota found on the fruit surface, or that leaves and unripe fruit are colonized by the same wind-borne microorganisms early in the growing season before fruit ripening and niche effects (see Glossary) cause them to diverge.

## The Vineyard Microenvironment in Flux: Temporal and Anthropogenic Effects

The prior sections examined general drivers of microbiome assembly in vineyards below- and aboveground. Site is a primary driver of this process (as a combination of different environmental effects), selecting the locally available pool of microbiota as well as the routes of transmission from source to sink. Plant factors (genotype and compartment) then influence the assembly of microbiomes at different plant sites. However, none of this occurs in a vacuum and the surrounding environmental conditions are constantly in flux; thus, seasonal factors, such as environmental, plant ripening, and anthropogenic (viticultural management) effects further complicate the processes of assembly.

### Phyllosphere Seasonality

The vineyard is a system undergoing cyclical change, with a predictable seasonal trajectory for plant phenological development and the associated microbiome. Grapevines are dormant in the winter, and shoots with vegetative structures emerge in the spring, preceding flowers and subsequent fruit. The structure and function of leaves and fruit in particular change through the season. Season is a major driver of microbiome structure because the morphological changes in host plants exert pressures on microbiota, in conjunction with community succession dynamics and microbe-microbe interactions. These effects have been noted in the bacterial communities of annuals (Maignien et al., 2014; Copeland et al., 2015), and in perennials including grapevines (Martins et al., 2012; Wagner et al., 2016; Grady et al., 2019). Specifically, seasonality and plant compartment distinction have been noted in microbiomes on fruit and leaves in grapevines (Leveau and Tech, 2011; Martins et al., 2012, 2013; Morrison-Whittle and Goddard, 2015; Zarraonaindia et al., 2015).

A typical succession of microbiota occurs through ripening on the fruit surface (Barata et al., 2012a,b; Bisson et al., 2017). α-Diversity decreases and β-diversity (the comparative diversity between samples in an environment) increases as the growing season progresses, suggesting that microbiomes are gradually restructured through host-microbe interactions, such as with sugar exudates on grape surfaces stimulating the proliferation of yeasts. Similar changes occur on grapevine leaf surfaces during the growing season (Pinto et al., 2014), though it is unclear how much this is driven by phenology vs. management effects.

The arc of these changes shows interannual regularity, but site-specific effects remain detectable. Often, microbial communities in the phyllosphere are similar at the onset of the growing season before diverging. These effects are further impacted by farming practices (Martins et al., 2012, 2014). Further, microbe-microbe interactions also play a role in phyllosphere microbiome assembly. Environmental and host genotype factors interact to enrich specific “hub” microbes that modulate further community membership through microbe-microbe interactions (Agler et al., 2016). Early colonizers can modulate the environment by altering the physical environment of the plant surface or by promoting secondary metabolites that other microbiota can utilize, thereby shaping the developing community structure (Bodenhausen et al., 2014; Chappell and Fukami, 2018; Toju et al., 2018). Taken together, these findings suggest interannual seasonal trajectory of phyllosphere communities in plants generally, including grapevines. The predictable features of this are a decrease in α-diversity, and an increase in β-diversity between hosts.

### Grape Berry Microbiota: Effects of Ripening

The grape berry surface contains exudates, including sugars, mineral nutrients, and organic acids, the composition of which change across ripening. Exudates and cuticle waxes change in composition and thickness as cracks in the berry surface begin to occur at ripeness. These changes alter the susceptibility of the berry to pathogens and likely play a role in the aforementioned seasonal changes in microbiomes. Both fungal and bacterial communities increase in population size as ripening progresses and the communities on fruit change in composition, and these effects are modulated by farming practices.

Yeasts are in the minority on the grape surface, present between 10 and 10^3^ cfu/g on immature grapes and 10^4^–10^6^ cfu/g on ripe fruit (Fleet, 2003), with damage inducing a log fold change in abundance (Barata et al., 2012a; Pinto et al., 2014; Bisson et al., 2017). Early on, Basidiomycetous taxa, including *Aureobasidium*, *Cryptococcus*, and *Rhodotorula* dominate, and the carposphere resembles the phyllosphere of leaves (Barata et al., 2012b) before giving way to Ascomycetous yeasts, like *Hanseniaspora*, *Metschnikowia*, and *Picha* through maturation. This trend is evident all over the world, reflecting standard ecological succession at a certain taxonomic level (Bisson et al., 2017). These yeasts are in the minority compared with filamentous fungi, like *Aspergillus*, *Alternaria*, and *Fusarium*, and diverse bacteria (Bisson et al., 2017). Cultivable fungal diversity and richness increase throughout ripening and appear to be impacted by agricultural practices (Martins et al., 2014).

Diverse bacteria populate the ripening berry surface, including ubiquitous soil-borne *Bacillus* species and taxa typically found in the phyllosphere (Barata et al., 2012b). Contrary to Bisson et al. (2017), Barata et al. (2012a,b) report that bacteria exist in lesser numbers and that lactic acid bacteria are typically found at ~10^2^ CFU/g while acetic acid bacteria range from 10 to 10^6^ CFU/g in damaged fruit. Bacteria also exhibit typical ecological succession through ripening at high taxonomic resolution, characterized by a gradual decrease in abundance of Gram-negative organisms like *Pseudomonas* spp. while Gram-positive bacteria like *Micrococcus* increase in abundance during ripening (Martins et al., 2012).

If berries are damaged, the proliferation of *Ascomycetes*, like *Pichia*, *Zygosaccharomyces*, and *Torulaspora*, and bacteria like *Gluconobacter* and *Acetobacter* occurs (Nisiotou et al., 2007; Barata et al., 2012b). Damaged fruit not only results in an increase in overall abundance, but also an increase in the diversity of taxa and community remodeling (Barata et al., 2012b). The changes associated with ripening, particularly the change in the availability of substrates likely alters the microbial composition of the fruit surface.

### A Brief Word on Management Practices

Human intervention also impacts microbial transmission and assembly within vineyards, impacting the many sources and vectors discussed earlier in this review, and thus deserves brief mention although a complete inspection is out of scope of this review. Human impacts can be intentional and direct, such as through application of fungicides. Anthropogenic activity could also exert indirect effects on microbial transmission through insecticide use, canopy management, winery workers entering vineyards and acting as vectors, and other practices that impact microbial vectors and microclimate. While various studies have described differences between vineyards under different management practices (Bevivino et al., 2014; Morrison-Whittle et al., 2017; Castaneda et al., 2018; Giraldo-Perez et al., 2021), existing data on management effects are limited by lack of control for block effects and site variation in most studies. Future studies should control for block and site effects when examining management practices. Similarly, studies of fruit or must microbiome site or cultivar effects should control for ripening and management effects due to their demonstrated effect on composition of the associated microbial communities.

## Conclusion

The microbial ecosystems within vineyards exert critical influences on grapevine health and wine quality, and hence understanding both the sources of microbiota within vineyards and effectors on community assembly is important for addressing various challenges to winegrowing. Vineyard location, microenvironment, cultivar, management, and seasonality all clearly play some role, but it is challenging to parse their relative contributions, as seen from existing studies of commercial vineyards. The unique confluence of local and regional environment, soil properties, grapevine cultivar, surrounding plants and animals, and human interventions shape the microbial diversity found within vineyards, and are potentially features that contribute to the uniqueness of wines from different sites.

The precise sources and vectors of microbiota within vineyards are not fully established, but soil, local plants and animals, weather, and human practices are clearly involved in shaping annual patterns of microbial assembly within individual vineyards. Site-specific edaphic properties exert selective pressures to guide microbiome assembly in soils, which may serve as an important reservoir for transmission of microbiota to above-ground plant organs, as well as drive microbial interactions with vines within the rhizosphere. Landscape-scale features including neighboring plant life (including grapevines themselves) likely shape the potential microbial pool available locally for deposition on vineyard surfaces. Landscape connectivity may similarly contribute to sources of microbiota found in vineyards, and regional and local weather patterns are clearly involved in microbial dispersion, whether the initial source is soil or other plants. These factors together contribute to inter-annually stable seeding events to initiate microbiome re-assembly on grapevines seasonally, which then changes through the growing season, influenced by cultivar effects and cyclical effects of phenology, weather, and viticultural management practices. Sources and stability of microbiota can be argued to be inter-annually regular features of specific vineyards, playing a role in the unique challenges of each site. Further work is required to understand which of these proposed sources of microbiota in vineyards are reliable features of the vineyard ecosystem and have the potential to inform winegrowing decisions on a region and site-specific basis.

## Author Contributions

RG and NB wrote the article with contributions from all authors. All authors contributed to the article and approved the submitted version.

### Conflict of Interest

The authors declare that the research was conducted in the absence of any commercial or financial relationships that could be construed as a potential conflict of interest.

## Figures and Tables

**Table 1:** 

α-Diversity	The measurement of diversity within a single sample, e.g., the number or distribution of species or other taxonomic units.
β-Diversity	The measurement of diversity (dis)similarity between two samples, e.g., heterogeneity in community composition or structure.
Biogeography	Refers both to the spatial distribution of biodiversity and to its study.
Carposphere	The microbial habitats present in and on fruit.
Dispersal limitation	The hypothesis that species dispersal is limited by spatial distance. In other words, the likelihood of a taxon occurring at a certain site changes with distance.
Environmental filtering	Filtering of taxa by the abiotic or microbial constraints of a specific environment.
Hub taxa	Taxa that are selected for by plant genotype or abiotic factors, which then have a disproportionate impact on community structure through microbe-microbe interactions.
Niche effects	The collection of environmental factors that exert selective pressures on (microbial) communities within a specific ecosystem, driving community assembly at that site.
Microbial terroir	The connection between regional variation in microbial profiles and food characteristics. The first complete definition of this compound term in the scientific literature was provided in 2014 (Bokulich et al., 2014). The term was used as early as 2012 with regard to wine (Bokulich et al., 2012b) and beer fermentations (Bokulich et al., 2012a), and preceded by earlier discussions of microbial biodiversity and terroir (Gayevskiy and Goddard, 2012). The idea that microbial activity contributes to terroir is much older, however, rooted in the empirical observations of winegrowers, e.g., that inoculation suppresses terroir characteristics.
Microbiome	A complex term that encompasses the multi-species microbial communities present in an ecosystem (microbiota), their chemical constituents and products, their functional activities, and their interactions with each other and their environment (Berg et al., 2020)
Microbiota	Refers to a multi-species microbial community, all microorganisms present in an ecosystem or sample.
Phyllosphere	The microbial habitats present in and on plant leaves (Ruinen, 1956), but more generally used to refer to all above-ground plant organs as a habitat for microbiota.
Terroir	Regional variations in food or crop phenotypes (e.g., grape and wine properties). Biological correlates for this phenomenon in winegrapes include geographic and intra-vineyard differences in phenolic compounds (Brillante et al., 2017; Urvieta et al., 2021), micronutrients (Tanabe et al., 2020), sensory-active compounds (Scarlett et al., 2014), and grapevine gene expression profiles (Anesi et al., 2015). The full connectivity between place and resultant wine remains to be elucidated, to match the cultural understanding of the phenomenon.
